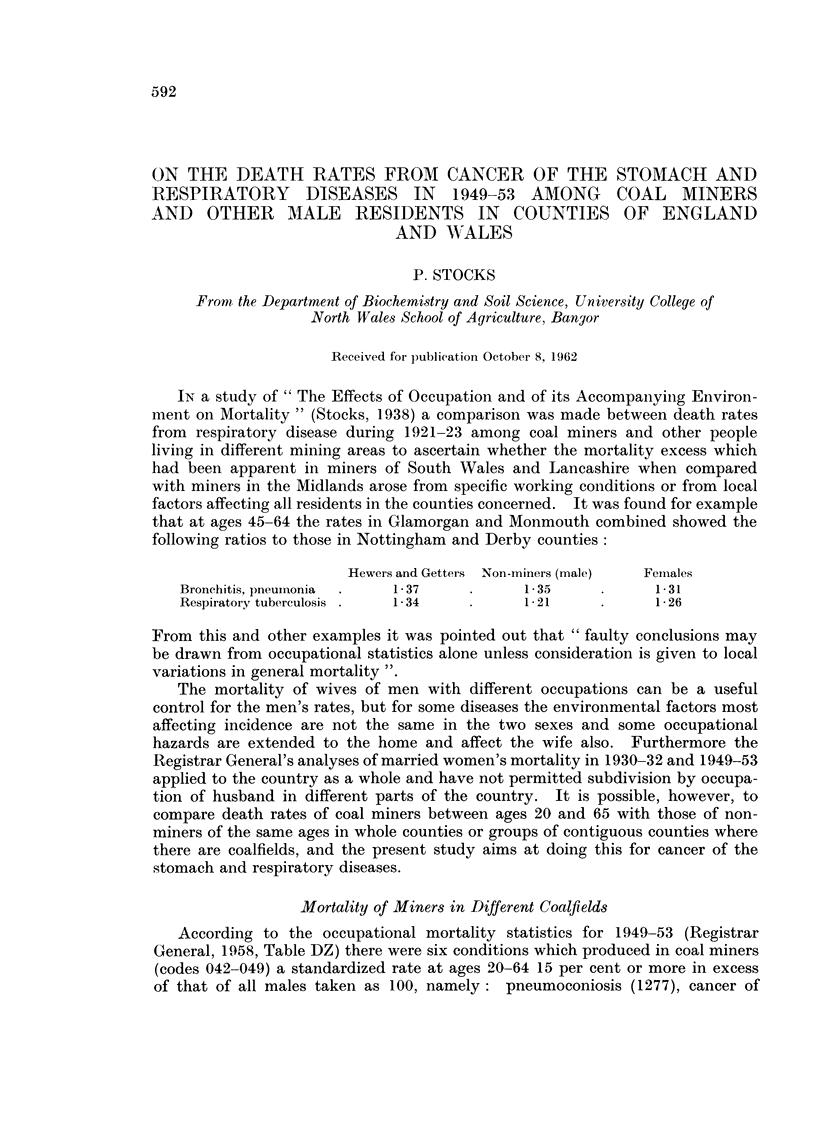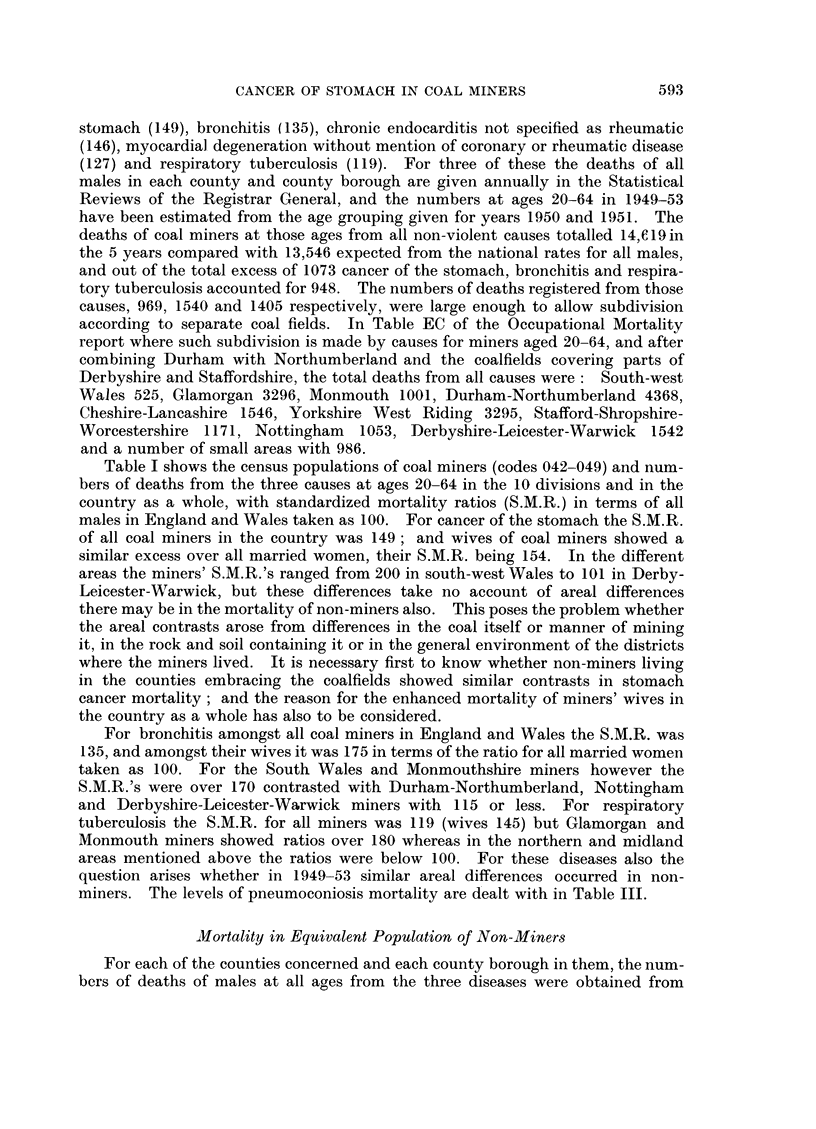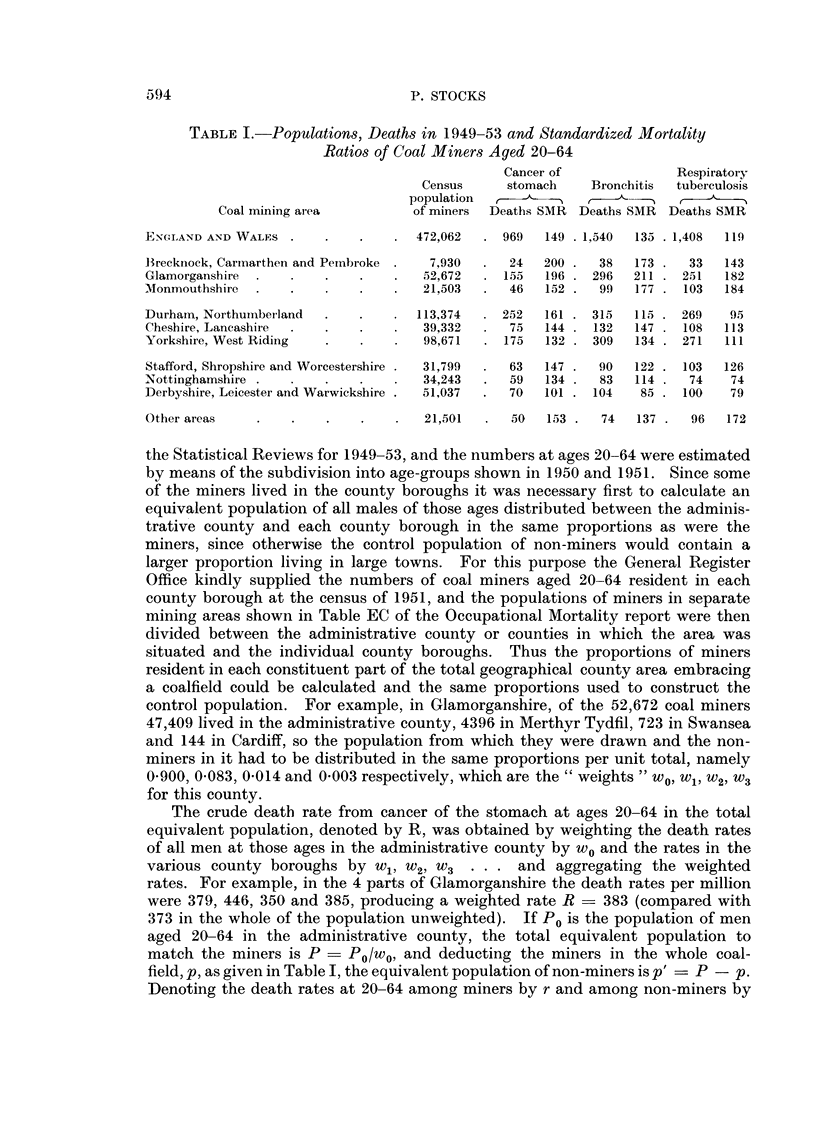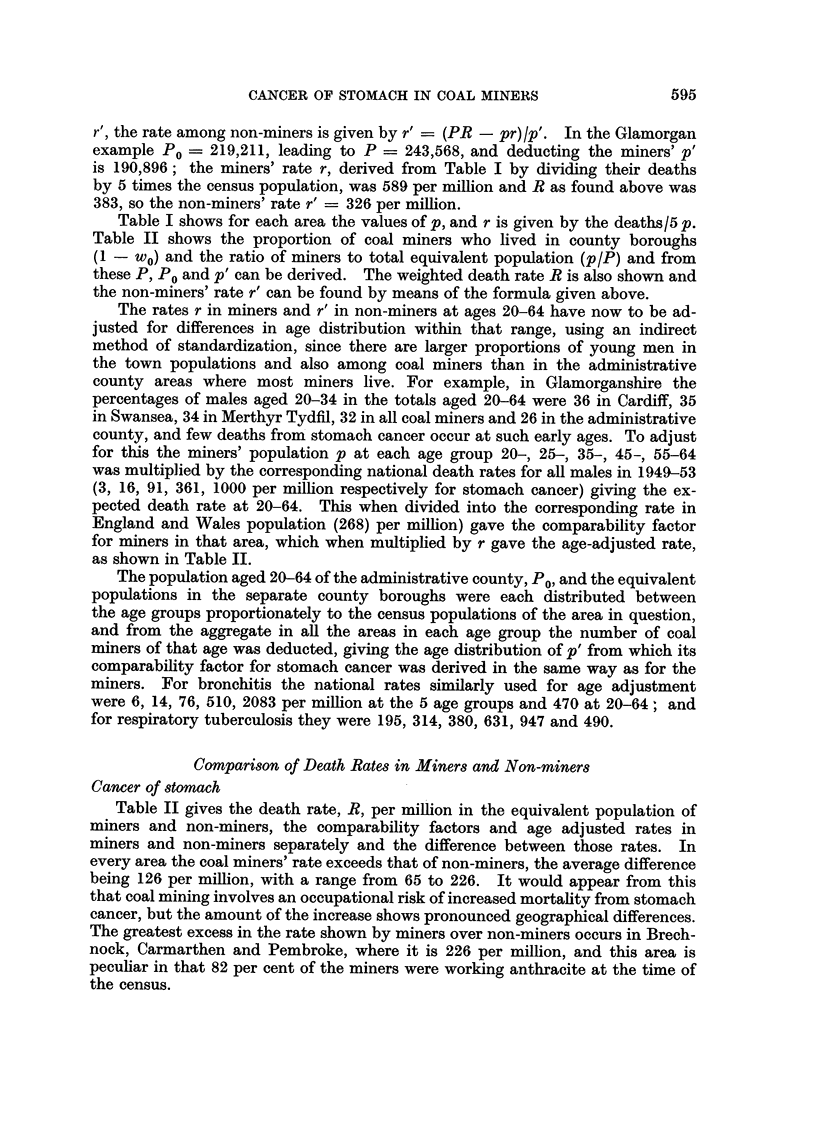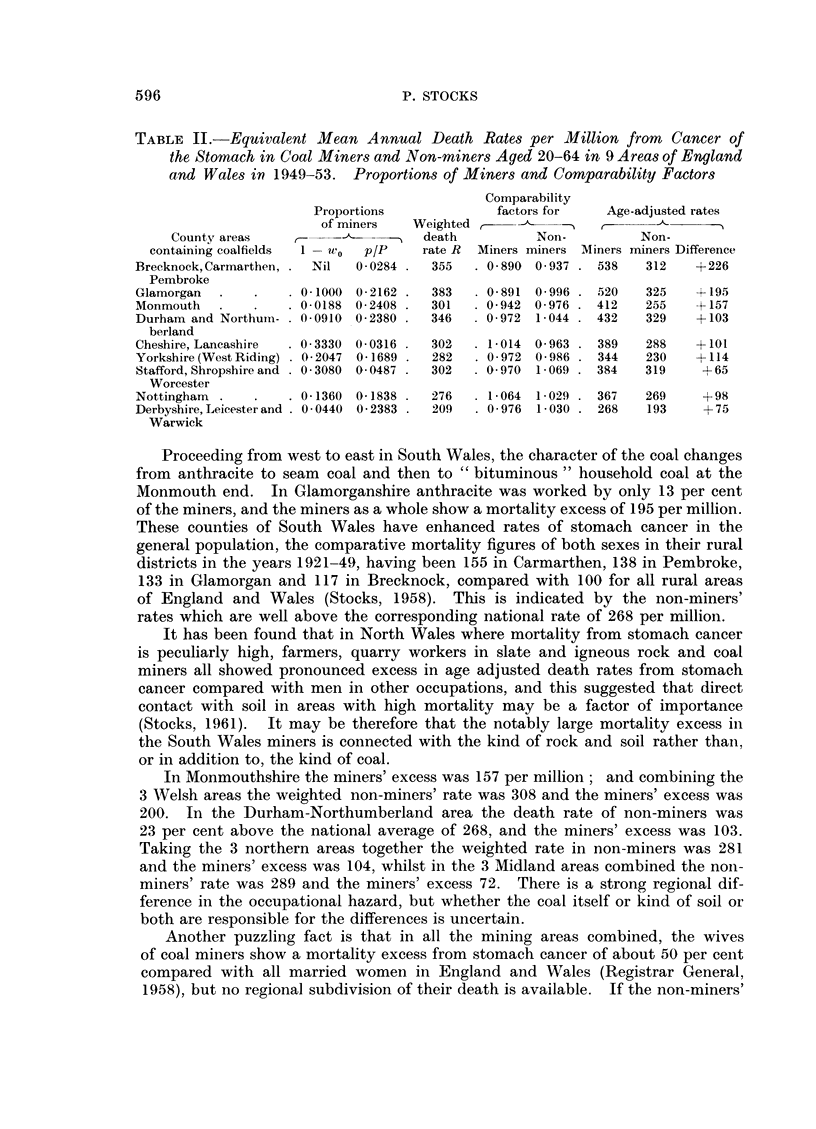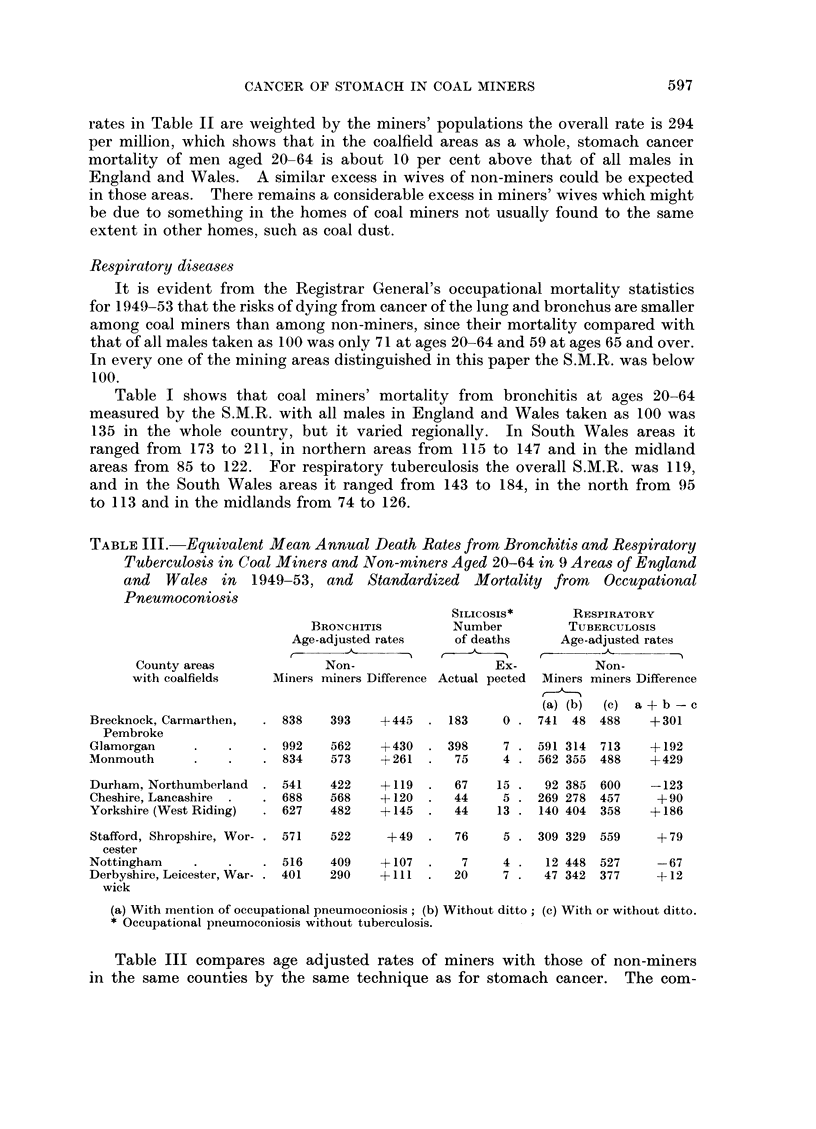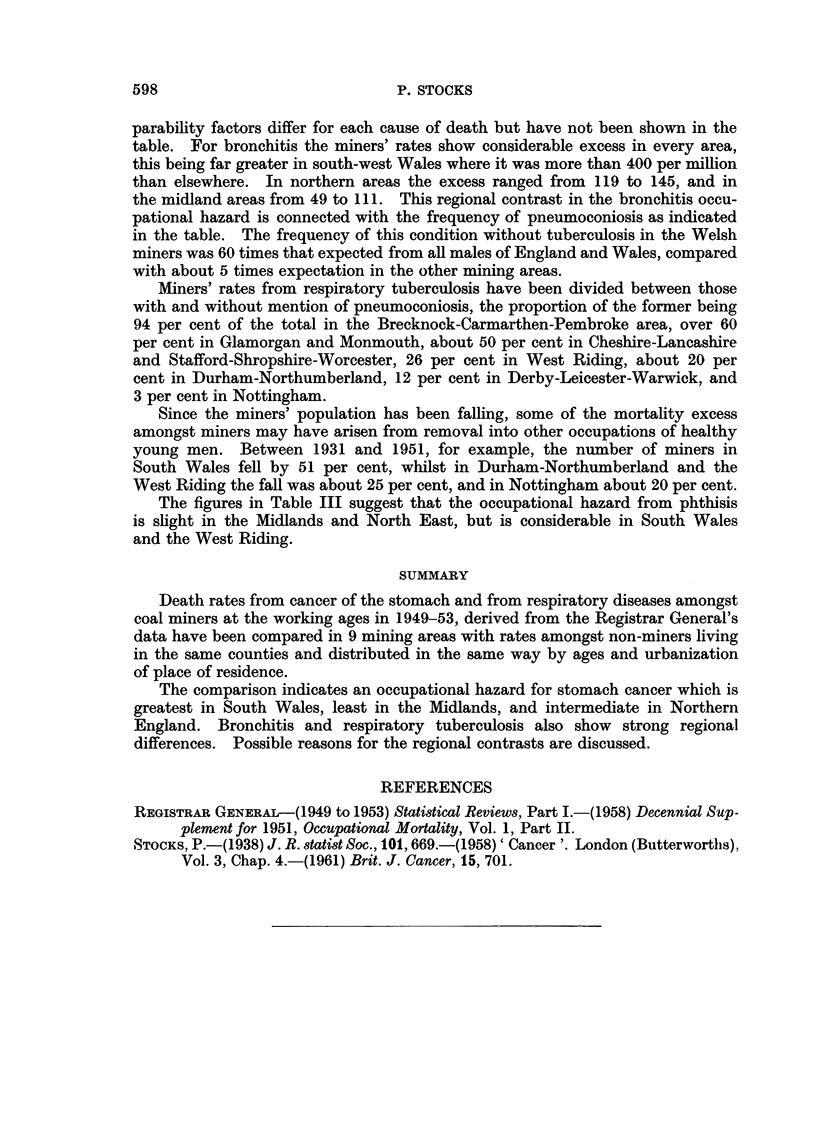# On the Death Rates from Cancer of the Stomach and Respiratory Diseases in 1949-53 among coal miners and other male residents in counties of England and Wales

**DOI:** 10.1038/bjc.1962.69

**Published:** 1962-12

**Authors:** P. Stocks


### ON THE DEATH RATES FROM CANCER OF THE STOMACH AND RESPIRATORY DISEASES IN 1949–53 AMONG COAL MINERS AND OTHER MALE RESIDENTS IN COUNTIES OF ENGLAND AND WALES

P. STOCKS

*From the Department of Biochemistry and Soil Science, University College of North Wales School of Agriculture, Bangor*

Received for publication October 8, 1962

IN a study of " The Effects of Occupation and of its Accompanying Environment on Mortality " (Stocks, 1938) a comparison was made between death rates from respiratory disease during 1921–23 among coal miners and other people living in different mining areas to ascertain whether the mortality excess which had been apparent in miners of South Wales and Lancashire when compared with miners in the Midlands arose from specific working conditions or from local factors affecting all residents in the counties concerned. It was found for example that at ages 45–64 the rates in Glamorgan and Monmouth combined showed the following ratios to those in Nottingham and Derby counties :

| | Hewers and Getters | Non-miners (male) | Females |
|---|---|---|---|
| Bronchitis, pneumonia | 1·37 | 1·35 | 1·31 |
| Respiratory tuberculosis | 1·34 | 1·21 | 1·26 |

From this and other examples it was pointed out that " faulty conclusions may be drawn from occupational statistics alone unless consideration is given to local variations in general mortality ".

The mortality of wives of men with different occupations can be a useful control for the men's rates, but for some diseases the environmental factors most affecting incidence are not the same in the two sexes and some occupational hazards are extended to the home and affect the wife also. Furthermore the Registrar General's analyses of married women's mortality in 1930–32 and 1949–53 applied to the country as a whole and have not permitted subdivision by occupation of husband in different parts of the country. It is possible, however, to compare death rates of coal miners between ages 20 and 65 with those of non-miners of the same ages in whole counties or groups of contiguous counties where there are coalfields, and the present study aims at doing this for cancer of the stomach and respiratory diseases.

### *Mortality of Miners in Different Coalfields*

According to the occupational mortality statistics for 1949–53 (Registrar General, 1958, Table DZ) there were six conditions which produced in coal miners (codes 042–049) a standardized rate at ages 20–64 15 per cent or more in excess of that of all males taken as 100, namely : pneumoconiosis (1277), cancer of stomach (149), bronchitis (135), chronic endocarditis not specified as rheumatic (146), myocardial degeneration without mention of coronary or rheumatic disease (127) and respiratory tuberculosis (119). For three of these the deaths of all males in each county and county borough are given annually in the Statistical Reviews of the Registrar General, and the numbers at ages 20–64 in 1949–53 have been estimated from the age grouping given for years 1950 and 1951. The deaths of coal miners at those ages from all non-violent causes totalled 14,619 in the 5 years compared with 13,546 expected from the national rates for all males, and out of the total excess of 1073 cancer of the stomach, bronchitis and respiratory tuberculosis accounted for 948. The numbers of deaths registered from those causes, 969, 1540 and 1405 respectively, were large enough to allow subdivision according to separate coal fields. In Table EC of the Occupational Mortality report where such subdivision is made by causes for miners aged 20–64, and after combining Durham with Northumberland and the coalfields covering parts of Derbyshire and Staffordshire, the total deaths from all causes were : South-west Wales 525, Glamorgan 3296, Monmouth 1001, Durham-Northumberland 4368, Cheshire-Lancashire 1546, Yorkshire West Riding 3295, Stafford-Shropshire-Worcestershire 1171, Nottingham 1053, Derbyshire-Leicester-Warwick 1542 and a number of small areas with 986.

Table I shows the census populations of coal miners (codes 042–049) and numbers of deaths from the three causes at ages 20–64 in the 10 divisions and in the country as a whole, with standardized mortality ratios (S.M.R.) in terms of all males in England and Wales taken as 100. For cancer of the stomach the S.M.R. of all coal miners in the country was 149 ; and wives of coal miners showed a similar excess over all married women, their S.M.R. being 154. In the different areas the miners' S.M.R.'s ranged from 200 in south-west Wales to 101 in Derby-Leicester-Warwick, but these differences take no account of areal differences there may be in the mortality of non-miners also. This poses the problem whether the areal contrasts arose from differences in the coal itself or manner of mining it, in the rock and soil containing it or in the general environment of the districts where the miners lived. It is necessary first to know whether non-miners living in the counties embracing the coalfields showed similar contrasts in stomach cancer mortality ; and the reason for the enhanced mortality of miners' wives in the country as a whole has also to be considered.

For bronchitis amongst all coal miners in England and Wales the S.M.R. was 135, and amongst their wives it was 175 in terms of the ratio for all married women taken as 100. For the South Wales and Monmouthshire miners however the S.M.R.'s were over 170 contrasted with Durham-Northumberland, Nottingham and Derbyshire-Leicester-Warwick miners with 115 or less. For respiratory tuberculosis the S.M.R. for all miners was 119 (wives 145) but Glamorgan and Monmouth miners showed ratios over 180 whereas in the northern and midland areas mentioned above the ratios were below 100. For these diseases also the question arises whether in 1949–53 similar areal differences occurred in non-miners. The levels of pneumoconiosis mortality are dealt with in Table III.

### *Mortality in Equivalent Population of Non-Miners*

For each of the counties concerned and each county borough in them, the numbers of deaths of males at all ages from the three diseases were obtained from

TABLE I.—*Populations, Deaths in* 1949–53 *and Standardized Mortality Ratios of Coal Miners Aged* 20–64

| Coal mining area | Census population of miners | Cancer of stomach Deaths | Cancer of stomach SMR | Bronchitis Deaths | Bronchitis SMR | Respiratory tuberculosis Deaths | Respiratory tuberculosis SMR |
|---|---|---|---|---|---|---|---|
| ENGLAND AND WALES | 472,062 | 969 | 149 | 1,540 | 135 | 1,408 | 119 |
| Brecknock, Carmarthen and Pembroke | 7,930 | 24 | 200 | 38 | 173 | 33 | 143 |
| Glamorganshire | 52,672 | 155 | 196 | 296 | 211 | 251 | 182 |
| Monmouthshire | 21,503 | 46 | 152 | 99 | 177 | 103 | 184 |
| Durham, Northumberland | 113,374 | 252 | 161 | 315 | 115 | 269 | 95 |
| Cheshire, Lancashire | 39,332 | 75 | 144 | 132 | 147 | 108 | 113 |
| Yorkshire, West Riding | 98,671 | 175 | 132 | 309 | 134 | 271 | 111 |
| Stafford, Shropshire and Worcestershire | 31,799 | 63 | 147 | 90 | 122 | 103 | 126 |
| Nottinghamshire | 34,243 | 59 | 134 | 83 | 114 | 74 | 74 |
| Derbyshire, Leicester and Warwickshire | 51,037 | 70 | 101 | 104 | 85 | 100 | 79 |
| Other areas | 21,501 | 50 | 153 | 74 | 137 | 96 | 172 |

the Statistical Reviews for 1949–53, and the numbers at ages 20–64 were estimated by means of the subdivision into age-groups shown in 1950 and 1951. Since some of the miners lived in the county boroughs it was necessary first to calculate an equivalent population of all males of those ages distributed between the administrative county and each county borough in the same proportions as were the miners, since otherwise the control population of non-miners would contain a larger proportion living in large towns. For this purpose the General Register Office kindly supplied the numbers of coal miners aged 20–64 resident in each county borough at the census of 1951, and the populations of miners in separate mining areas shown in Table EC of the Occupational Mortality report were then divided between the administrative county or counties in which the area was situated and the individual county boroughs. Thus the proportions of miners resident in each constituent part of the total geographical county area embracing a coalfield could be calculated and the same proportions used to construct the control population. For example, in Glamorganshire, of the 52,672 coal miners 47,409 lived in the administrative county, 4396 in Merthyr Tydfil, 723 in Swansea and 144 in Cardiff, so the population from which they were drawn and the non-miners in it had to be distributed in the same proportions per unit total, namely 0·900, 0·083, 0·014 and 0·003 respectively, which are the " weights " $w_0$, $w_1$, $w_2$, $w_3$ for this county.

The crude death rate from cancer of the stomach at ages 20–64 in the total equivalent population, denoted by R, was obtained by weighting the death rates of all men at those ages in the administrative county by $w_0$ and the rates in the various county boroughs by $w_1$, $w_2$, $w_3$ . . . and aggregating the weighted rates. For example, in the 4 parts of Glamorganshire the death rates per million were 379, 446, 350 and 385, producing a weighted rate $R = 383$ (compared with 373 in the whole of the population unweighted). If $P_0$ is the population of men aged 20–64 in the administrative county, the total equivalent population to match the miners is $P = P_0/w_0$, and deducting the miners in the whole coalfield, $p$, as given in Table I, the equivalent population of non-miners is $p' = P - p$. Denoting the death rates at 20–64 among miners by $r$ and among non-miners by

$r'$, the rate among non-miners is given by $r' = (PR - pr)/p'$. In the Glamorgan example $P_0 = 219{,}211$, leading to $P = 243{,}568$, and deducting the miners' $p'$ is 190,896; the miners' rate $r$, derived from Table I by dividing their deaths by 5 times the census population, was 589 per million and $R$ as found above was 383, so the non-miners' rate $r' = 326$ per million.

Table I shows for each area the values of $p$, and $r$ is given by the deaths/5 $p$. Table II shows the proportion of coal miners who lived in county boroughs $(1 - w_0)$ and the ratio of miners to total equivalent population $(p/P)$ and from these $P$, $P_0$ and $p'$ can be derived. The weighted death rate $R$ is also shown and the non-miners' rate $r'$ can be found by means of the formula given above.

The rates $r$ in miners and $r'$ in non-miners at ages 20–64 have now to be adjusted for differences in age distribution within that range, using an indirect method of standardization, since there are larger proportions of young men in the town populations and also among coal miners than in the administrative county areas where most miners live. For example, in Glamorganshire the percentages of males aged 20–34 in the totals aged 20–64 were 36 in Cardiff, 35 in Swansea, 34 in Merthyr Tydfil, 32 in all coal miners and 26 in the administrative county, and few deaths from stomach cancer occur at such early ages. To adjust for this the miners' population $p$ at each age group 20–, 25–, 35–, 45–, 55–64 was multiplied by the corresponding national death rates for all males in 1949–53 (3, 16, 91, 361, 1000 per million respectively for stomach cancer) giving the expected death rate at 20–64. This when divided into the corresponding rate in England and Wales population (268) per million) gave the comparability factor for miners in that area, which when multiplied by $r$ gave the age-adjusted rate, as shown in Table II.

The population aged 20–64 of the administrative county, $P_0$, and the equivalent populations in the separate county boroughs were each distributed between the age groups proportionately to the census populations of the area in question, and from the aggregate in all the areas in each age group the number of coal miners of that age was deducted, giving the age distribution of $p'$ from which its comparability factor for stomach cancer was derived in the same way as for the miners. For bronchitis the national rates similarly used for age adjustment were 6, 14, 76, 510, 2083 per million at the 5 age groups and 470 at 20–64; and for respiratory tuberculosis they were 195, 314, 380, 631, 947 and 490.

### *Comparison of Death Rates in Miners and Non-miners*

#### *Cancer of stomach*

Table II gives the death rate, $R$, per million in the equivalent population of miners and non-miners, the comparability factors and age adjusted rates in miners and non-miners separately and the difference between those rates. In every area the coal miners' rate exceeds that of non-miners, the average difference being 126 per million, with a range from 65 to 226. It would appear from this that coal mining involves an occupational risk of increased mortality from stomach cancer, but the amount of the increase shows pronounced geographical differences. The greatest excess in the rate shown by miners over non-miners occurs in Brecknock, Carmarthen and Pembroke, where it is 226 per million, and this area is peculiar in that 82 per cent of the miners were working anthracite at the time of the census.

TABLE II.—*Equivalent Mean Annual Death Rates per Million from Cancer of the Stomach in Coal Miners and Non-miners Aged* 20–64 *in* 9 *Areas of England and Wales in* 1949–53. *Proportions of Miners and Comparability Factors*

| County areas containing coalfields | Proportions of miners | | Weighted death rate $R$ | Comparability factors for | | Age-adjusted rates | | |
|---|---|---|---|---|---|---|---|---|
| | $1 - w_0$ | $p/P$ | | Miners | Non-miners | Miners | Non-miners | Difference |
| Brecknock, Carmarthen, Pembroke | Nil | 0·0284 | 355 | 0·890 | 0·937 | 538 | 312 | +226 |
| Glamorgan | 0·1000 | 0·2162 | 383 | 0·891 | 0·996 | 520 | 325 | +195 |
| Monmouth | 0·0188 | 0·2408 | 301 | 0·942 | 0·976 | 412 | 255 | +157 |
| Durham and Northumberland | 0·0910 | 0·2380 | 346 | 0·972 | 1·044 | 432 | 329 | +103 |
| Cheshire, Lancashire | 0·3330 | 0·0316 | 302 | 1·014 | 0·963 | 389 | 288 | +101 |
| Yorkshire (West Riding) | 0·2047 | 0·1689 | 282 | 0·972 | 0·986 | 344 | 230 | +114 |
| Stafford, Shropshire and Worcester | 0·3080 | 0·0487 | 302 | 0·970 | 1·069 | 384 | 319 | +65 |
| Nottingham | 0·1360 | 0·1838 | 276 | 1·064 | 1·029 | 367 | 269 | +98 |
| Derbyshire, Leicester and Warwick | 0·0440 | 0·2383 | 209 | 0·976 | 1·030 | 268 | 193 | +75 |

Proceeding from west to east in South Wales, the character of the coal changes from anthracite to seam coal and then to " bituminous " household coal at the Monmouth end. In Glamorganshire anthracite was worked by only 13 per cent of the miners, and the miners as a whole show a mortality excess of 195 per million. These counties of South Wales have enhanced rates of stomach cancer in the general population, the comparative mortality figures of both sexes in their rural districts in the years 1921–49, having been 155 in Carmarthen, 138 in Pembroke, 133 in Glamorgan and 117 in Brecknock, compared with 100 for all rural areas of England and Wales (Stocks, 1958). This is indicated by the non-miners' rates which are well above the corresponding national rate of 268 per million.

It has been found that in North Wales where mortality from stomach cancer is peculiarly high, farmers, quarry workers in slate and igneous rock and coal miners all showed pronounced excess in age adjusted death rates from stomach cancer compared with men in other occupations, and this suggested that direct contact with soil in areas with high mortality may be a factor of importance (Stocks, 1961). It may be therefore that the notably large mortality excess in the South Wales miners is connected with the kind of rock and soil rather than, or in addition to, the kind of coal.

In Monmouthshire the miners' excess was 157 per million ; and combining the 3 Welsh areas the weighted non-miners' rate was 308 and the miners' excess was 200. In the Durham-Northumberland area the death rate of non-miners was 23 per cent above the national average of 268, and the miners' excess was 103. Taking the 3 northern areas together the weighted rate in non-miners was 281 and the miners' excess was 104, whilst in the 3 Midland areas combined the non-miners' rate was 289 and the miners' excess 72. There is a strong regional difference in the occupational hazard, but whether the coal itself or kind of soil or both are responsible for the differences is uncertain.

Another puzzling fact is that in all the mining areas combined, the wives of coal miners show a mortality excess from stomach cancer of about 50 per cent compared with all married women in England and Wales (Registrar General, 1958), but no regional subdivision of their death is available. If the non-miners'

rates in Table II are weighted by the miners' populations the overall rate is 294 per million, which shows that in the coalfield areas as a whole, stomach cancer mortality of men aged 20–64 is about 10 per cent above that of all males in England and Wales. A similar excess in wives of non-miners could be expected in those areas. There remains a considerable excess in miners' wives which might be due to something in the homes of coal miners not usually found to the same extent in other homes, such as coal dust.

*Respiratory diseases*

It is evident from the Registrar General's occupational mortality statistics for 1949–53 that the risks of dying from cancer of the lung and bronchus are smaller among coal miners than among non-miners, since their mortality compared with that of all males taken as 100 was only 71 at ages 20–64 and 59 at ages 65 and over. In every one of the mining areas distinguished in this paper the S.M.R. was below 100.

Table I shows that coal miners' mortality from bronchitis at ages 20–64 measured by the S.M.R. with all males in England and Wales taken as 100 was 135 in the whole country, but it varied regionally. In South Wales areas it ranged from 173 to 211, in northern areas from 115 to 147 and in the midland areas from 85 to 122. For respiratory tuberculosis the overall S.M.R. was 119, and in the South Wales areas it ranged from 143 to 184, in the north from 95 to 113 and in the midlands from 74 to 126.

TABLE III.—*Equivalent Mean Annual Death Rates from Bronchitis and Respiratory Tuberculosis in Coal Miners and Non-miners Aged* 20–64 *in* 9 *Areas of England and Wales in* 1949–53, *and Standardized Mortality from Occupational Pneumoconiosis*

| County areas with coalfields | Bronchitis Age-adjusted rates | | | Silicosis* Number of deaths | | Respiratory Tuberculosis Age-adjusted rates | | | |
|---|---|---|---|---|---|---|---|---|---|
| | Miners | Non-miners | Difference | Actual | Expected | Miners (a) | Miners (b) | Non-miners (c) | Difference a + b − c |
| Brecknock, Carmarthen, Pembroke | 838 | 393 | +445 | 183 | 0 | 741 | 48 | 488 | +301 |
| Glamorgan | 992 | 562 | +430 | 398 | 7 | 591 | 314 | 713 | +192 |
| Monmouth | 834 | 573 | +261 | 75 | 4 | 562 | 355 | 488 | +429 |
| Durham, Northumberland | 541 | 422 | +119 | 67 | 15 | 92 | 385 | 600 | −123 |
| Cheshire, Lancashire | 688 | 568 | +120 | 44 | 5 | 269 | 278 | 457 | +90 |
| Yorkshire (West Riding) | 627 | 482 | +145 | 44 | 13 | 140 | 404 | 358 | +186 |
| Stafford, Shropshire, Worcester | 571 | 522 | +49 | 76 | 5 | 309 | 329 | 559 | +79 |
| Nottingham | 516 | 409 | +107 | 7 | 4 | 12 | 448 | 527 | −67 |
| Derbyshire, Leicester, Warwick | 401 | 290 | +111 | 20 | 7 | 47 | 342 | 377 | +12 |

(a) With mention of occupational pneumoconiosis ; (b) Without ditto ; (c) With or without ditto.
* Occupational pneumoconiosis without tuberculosis.

Table III compares age adjusted rates of miners with those of non-miners in the same counties by the same technique as for stomach cancer. The comparability factors differ for each cause of death but have not been shown in the table. For bronchitis the miners' rates show considerable excess in every area, this being far greater in south-west Wales where it was more than 400 per million than elsewhere. In northern areas the excess ranged from 119 to 145, and in the midland areas from 49 to 111. This regional contrast in the bronchitis occupational hazard is connected with the frequency of pneumoconiosis as indicated in the table. The frequency of this condition without tuberculosis in the Welsh miners was 60 times that expected from all males of England and Wales, compared with about 5 times expectation in the other mining areas.

Miners' rates from respiratory tuberculosis have been divided between those with and without mention of pneumoconiosis, the proportion of the former being 94 per cent of the total in the Brecknock-Carmarthen-Pembroke area, over 60 per cent in Glamorgan and Monmouth, about 50 per cent in Cheshire-Lancashire and Stafford-Shropshire-Worcester, 26 per cent in West Riding, about 20 per cent in Durham-Northumberland, 12 per cent in Derby-Leicester-Warwick, and 3 per cent in Nottingham.

Since the miners' population has been falling, some of the mortality excess amongst miners may have arisen from removal into other occupations of healthy young men. Between 1931 and 1951, for example, the number of miners in South Wales fell by 51 per cent, whilst in Durham-Northumberland and the West Riding the fall was about 25 per cent, and in Nottingham about 20 per cent.

The figures in Table III suggest that the occupational hazard from phthisis is slight in the Midlands and North East, but is considerable in South Wales and the West Riding.

### SUMMARY

Death rates from cancer of the stomach and from respiratory diseases amongst coal miners at the working ages in 1949–53, derived from the Registrar General's data have been compared in 9 mining areas with rates amongst non-miners living in the same counties and distributed in the same way by ages and urbanization of place of residence.

The comparison indicates an occupational hazard for stomach cancer which is greatest in South Wales, least in the Midlands, and intermediate in Northern England. Bronchitis and respiratory tuberculosis also show strong regional differences. Possible reasons for the regional contrasts are discussed.

### REFERENCES

Registrar General—(1949 to 1953) *Statistical Reviews*, Part I.—(1958) *Decennial Supplement for* 1951, *Occupational Mortality*, Vol. 1, Part II.

Stocks, P.—(1938) *J. R. statist Soc.*, **101**, 669.—(1958) ' Cancer '. London (Butterworths), Vol. 3, Chap. 4.—(1961) *Brit. J. Cancer*, **15**, 701.